# Prognosis of nonspecific interstitial pneumonia correlates with perivascular CD4+ T lymphocyte infiltration of the lung

**DOI:** 10.1186/s12890-015-0122-z

**Published:** 2015-10-24

**Authors:** Ling Qin, WenZe Wang, HongRui Liu, Yi Xiao, MingWei Qin, WenJie Zheng, JuHong Shi

**Affiliations:** 1 Division of internal Medicine, Peking Union Medical College Hospital, Chinese Academy of Medical Sciences & Peking Union Medical College, Beijing, 100730 China; 2 Division of Pathology, Peking Union Medical College Hospital, Chinese Academy of Medical Sciences & Peking Union Medical College, Beijing, 100730 China; 3 Division of Pulmonary Medicine, Peking Union Medical College Hospital, Chinese Academy of Medical Sciences & Peking Union Medical College, Beijing, 100730 China; 4 Division of Rheumatology, Peking Union Medical College Hospital, Chinese Academy of Medical Sciences & Peking Union Medical College, Beijing, 100730 China; 5 Division of Radiology, Peking Union Medical College Hospital, Chinese Academy of Medical Sciences & Peking Union Medical College, Beijing, 100730 China

**Keywords:** T lymphocytes, Nonspecific interstitial pneumonia, Immunohistochemistry, Pathology

## Abstract

**Background:**

Nonspecific interstitial pneumonia (NSIP) is characterized by interstitial infiltration of T lymphocytes, and subpopulations of these cells may be associated with the progression of fibrosis. However, few studies evaluate the correlation of prognosis with this characteristic. Therefore, we performed morphological and quantitative analyses of T lymphocytes in patients with NSIP and evaluated the relationship between T lymphocytes and prognosis.

**Methods:**

Immunohistochemistry was used to detect the presence of CD4+ and CD8+ T lymphocytes in 55 biopsies of patients with NSIP to determine the numbers of these T cell subpopulations in lymphoid follicles as well as in perivascular, interstitial, and peribronchial anatomical compartments. The relationship between CD4+ and CD8+ T lymphocyte populations and prognosis was analyzed.

**Results:**

The mean age of 55 patients was 48.9 ± 10.5 years, and 36 (65 %) of patients were women. All patients were followed for a mean duration of 46 ± 25 months. Thirteen (23.6 %) patients died during follow-up. Perivascular CD4+ lymphocyte infiltration (HR, 0.939; 95 % CI, 0.883–0.999; *p* = 0.048) was an independent risk factor for survival. Perivascular infiltrates of CD4+ T lymphocytes correlated with survival time (*r* = 0.270, *p* = 0.046). Patients with improved forced vital capacity survived longer and had higher numbers of CD4+ T lymphocytes that infiltrated perivascular tissue. The densities of CD4+ and CD8+ T lymphocytes infiltrating other tissues were not significantly associated with survival time.

**Conclusions:**

Perivascular infiltration of CD4+ T lymphocytes in patients with NSIP correlated with prognosis. The underlying mechanisms are unknown and require further studies.

**Electronic supplementary material:**

The online version of this article (doi:10.1186/s12890-015-0122-z) contains supplementary material, which is available to authorized users.

## Background

Interstitial lung diseases (ILDs) are generally characterized by the accumulation of inflammatory cells within the lung, followed by the progressive deposition of extracellular matrix and subsequent destruction of alveolar airspaces [1]. The precise role of inflammatory cells in the pathogenesis of ILD remains poorly understood [2–4], although evidence indicates that T lymphocytes play an important role in the initiation and development of pulmonary fibrosis [5, 6]. However, few studies evaluate the prognostic significance of T lymphocyte subsets in patients with nonspecific interstitial pneumonia (NSIP) [7]. Therefore, we hypothesized that patients with NSIP who respond successfully to corticosteroid therapy harbor different T lymphocyte subpopulations compared with patients who do not respond.

To test this hypothesis, we evaluated the distribution of T lymphocyte subsets in the lung tissues of patients with NSIP. Using immunohistochemistry, we quantified the T lymphocyte subsets present in lymphoid follicles as well as in perivascular, interstitial, and peribronchial regions and explored the relationship between prognosis and survival.

## Methods

### Study subjects and diagnostic criteria

Between April 2003 and December 2011, 97 patients from Peking Union Medical College Hospital (PUMCH) were diagnosed with NSIP according to analysis of lung biopsies. Fifty-five patients who completed follow-up by undergoing tests of pulmonary function tests and computed tomography (CT) of the chest were included in this study. NSIP was diagnosed according to the American Thoracic Society (ATS)/European Respiratory Society consensus classification [8, 9]. Patients were not treated with corticosteroids or other immunosuppressants before undergoing a lung biopsy. Connective tissue disease (CTD) was diagnosed according to the criteria of the American College of Rheumatology as follows: autoantibodies against the nucleus (ANA titer >1:320), rheumatoid factor, Sjögren’s-syndrome-related antigens SSA or SSB, Scl-70, Sm, anti-Jo-1, ribonucleoprotein, or cyclic citrullinated peptide [10–13]. Patients with NSIP were classified into the groups as follows: (1) Members of the CTD-NSIP group met the criteria of the American College of Rheumatology for CTD. (2) Members of the NSIP-Ab + group had at least one positive serologic test. (3) Members of the NSIP-Ab– group were autoantibody negative. We extracted clinical characteristics documented at the time of a patient’s first visit as follows: age, ethnicity, sex, symptoms (cough, dyspnea, or wheeze) at the time of lung biopsy, symptoms or signs of CTD, smoking status, physical examination findings, pulmonary function results, serologic results, and chest CT scan.

Informed consent to use medical records was obtained from every patient, their guardian, or both when the patient was admitted to the hospital. The PUMCH Institutional Review Board approved this study (reference number for ethics approval: 2012–10–312).

### Pulmonary physiological assessments

Spirometry, total lung capacity determined using plethysmography, forced vital capacity (FVC), and diffusing capacity of the lung for carbon monoxide were measured according to ATS recommendations [14–16], and the results are expressed as the percentage of predicted normal values. Prognosis was described according to FVC 1 year after steroid therapy. We classified patients as follows: FVC-group 1, improvement of FVC >10 %; FVC-group 2, improvement of FVC between 10 % and −10 %; and FVC-group 3, FVC reduced greater than −10 % [17–20].

### High-resolution CT

All patients underwent high-resolution CT (HRCT) of the chest upon initial evaluation. A radiologist who specializes in diffuse parenchymal lung disease reviewed the CT scans. The extent and prevalence of abnormalities were measured in the areas as follows: (1) from the lung apex to the carina, (2) from the carina to the upper pulmonary vein, and (3) from the upper pulmonary vein to the base of the lung. HRCT images were assessed to determine the extent of parenchymal abnormalities, including ground-glass opacity (GGO), reticulation, honeycombing, consolidation, and emphysema. The extent of these abnormalities was determined using a 10 % scale for six zones [21]. Prognosis is described according to the manifestations detected using CT 1 year after steroid therapy. Patients with NSIP were classified as follows: CT-group 1, improvement of GGO and reticular opacity >50 %; group, CT-group 2, lesions reduced between 30 % and 50 %, and CT-group 3, lesions reduced by <30 % [21–23].

### Histology of lung tissue

Two experienced pathologists reviewed independently the lung biopsy specimens, and the histologic pattern was assigned according to the consensus opinion. The pathologists were unaware of the patients’ clinical information. The histological patterns of the 55 patients were classified further, according to criteria proposed by Katzenstein and Fiorelli [24], as cellular, mixed, or fibrotic. Small airways were defined as those with an internal diameter <2 mm without cartilage in the airway walls [25]. Small blood vessels were defined as those with an internal diameter ≤100 μm [26].

### Immunohistochemical analysis of CD4 and CD8 expression in lung tissue

Lung biopsy specimens were fixed in 10 % neutral-buffered formalin, cut into slices, embedded in paraffin, and cut into 4-μm-thick sections. Paraffin sections were reacted anti-CD4 (clone SP35, Zeta), and anti-CD8 (clone EP1150, Zeta) antibodies using the labeled-streptavidin-biotin complex method. The sections were deparaffinized, rehydrated with Tris-Buffered Saline (TBS) (0.005 M Tris, 0.15 M NaCl, pH 7.6) for 10 min, treated with 3 % hydrogen peroxide for 5 min to inhibit endogenous peroxidase activity, washed in TBS, and incubated with primary antibodies for 1 h. Immunohistochemical analyses of CD4 and CD8 expression were performed using the same tissue sections.

**Fig. 1:**
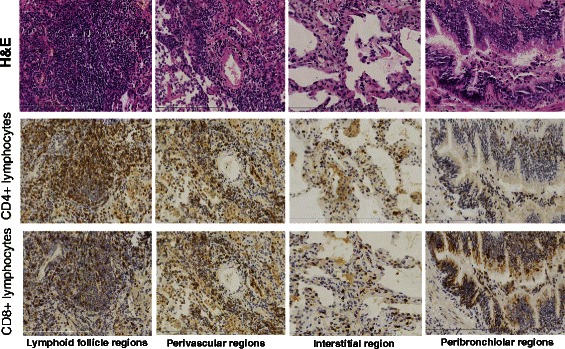
Distribution of T lymphocytes in different regions of lung tissue: CD4+ and CD8 + T lymphocytes decreased gradually in order of Lymphoid follicle, perivascular, interstitial and peribronchiolar regions. Positively stained of CD4+ and CD8 + T lymphocytes with dark brown staining

The numbers of dark-brown cells were determined using a NanoZoomer 2.0-RS Slide Scanning System (Hamamatsu Photonics KK; Japan Tokyo) and an Anymicro DSS Pro Image Analysis System (Yu Tian Shi Ji Wei Ye INC; Beijing, China). The follicles as well as perivascular, interstitial, and peribronchial regions were analyzed. The images of CD4+ and CD8+ T lymphocytes were obtained from the same region of the slide. At least six high-power fields (magnification × 200; analysis area, approximately 0.162 mm^2^) were randomly selected for each region and used to count the stained cells (Fig. 1).

### Treatment and follow up

All patients received a standard initial course of oral prednisone, starting at 0.5 mg/kg/d for 1 month that was tapered every 3 weeks to 5–7.5 mg/d (10 % decrease of the initial dose every 3 weeks), and maintained at 5–7.5 mg/d. Patients were treated for 12–18 months. Cyclophosphamide was administered concurrently with steroid therapy at an oral dose of 100 mg/d administered for 3–6 months to patients diagnosed with CTD-NSIP. Patients underwent routine examinations every 3–6 months after lung biopsy and then once each year at the Interstitial Lung Disease Clinic of PUMCH.

### Statistical analysis

All values are expressed as the mean ± standard deviation (SD). Normally distributed data were evaluated using the *t* test. The Wilcoxon and Kruskal–Wallis tests were used to compare two or more non-normally distributed values, respectively. Fisher’s exact test was used to determine differences between groups. Correlation coefficients were calculated using the Spearman rank method. Cox multivariate regression analysis was used to evaluate risk factors of survival. The times to overall survival were calculated using the Kaplan–Meier method. The probability value was obtained using 2-sided tests, and statistical significance was defined as *p* < 0.05. SPSS 15.0 (SPSS for Windows, version 15.0; SPSS Inc., Chicago, IL, USA) was used for statistical analysis.

## Results and discussion

### Clinical features and laboratory findings

The clinical, radiological, and physiological measurements obtained at the time of the initial visit are shown in Table 1. The mean age of 55 patients was 48.9 ± 10.5 years (range, 23–68 years), and 36 patients (65 %) were women. There were 47 nonsmokers, six former smokers, and two current smokers. The mean duration of respiratory symptoms from onset to lung biopsy was 6 months (20.2 ± 50.9 months; range, 0.8–360 months). All patients were followed for a mean duration of 64.3 ± 26.7 months (range, 14–120 months).

**Table 1:** Clinical features and laboratory findings of patients with NSIP

	NSIP (*n* = 55)
Clinical manifestations, N (%)	
Dyspnea	40 (72.7)
Cough	47(85.5)
Chest pain	4(7.3)
Dry eyes or dry mouth	3(5.5)
Fever	7 (12.7)
Arthralgia	9(16.4)
Skin rash	6(10.9)
Raynaud’s phenomenon	3(5.5)
Weight loss	4(7.3)
Crackles	41(74.6)
Clubbing	21(38.2)
Laboratory tests	
ESR, mm/h	21.2 ± 19.2
PaO_2_, mmHg	77.8 ± 12.9
PCO_2_, mmHg	37.9 ± 8.4
Serological results, N (%)	
Anti-nuclear antibody	14 (25.5)
Anti-SSA antibody	3 (5.5)
Anti-neutrophil cytoplasmic antibody	3 (5.5)
Anti-Jo-1 antibody	1 (1.8)
Anti-Scl-70 antibody	1 (1.8)
PFT	
TLC, % predicted	76.8 ± 14.1
DLCO, % predicted	56.4 ± 16.8
Baseline chest CT findings, N (%)	
Ground glass opacity	34 (61.8)
Patchy opacity	40 (72.7)
Irregular reticular opacity	35 (63.6)
Traction bronchiectasis	17 (30.9)
Pleural thickness	5 (14.6)

The patients were diagnosed as follows: 21 with CTD-NSIP (including seven with polymyositis/dermatomyositis; seven with rheumatoid arthritis; four with of Sjögren syndrome; two with systemic sclerosis, and one with microscopic polyangiitis), 14 with NSIP-Ab (+), and 20 with NSIP-Ab (−). There were no differences among the three groups according to age, sex, cough, and dyspnea. Symptoms of arthralgia, Raynaud’s phenomenon, skin rash, and dry eye or mouth were highly associated with the CTD-NSIP and NSIP-Ab + groups compared with the NSIP-Ab- group (Table 2). No differences among the three groups were noted in arterial blood gas and HRCT findings. Abnormalities of pulmonary function were similar among the three groups and were characterized by restrictive defects with impairment of diffusion (Table 3). During follow-up, three patients (15 %) with cellular NSIP died (two from lung infections, one from liver failure), and six (21 %) and four (80 %) patients with mixed or fibrotic NSIP, respectively, died from progression of lung disease.

**Table 2:** Clinical features of patients classified as CTD-NSIP, NSIP-Ab (+), or NSIP-Ab (−)

Characteristics	CTD-NSIP (*n* = 21)	NSIP-Ab (+) (*n* = 14)	NSIP-Ab (−) (*n* = 20)	*p* value
Age (years)	50.48 ± 11.73	44.57 ± 8.81	50.50 ± 9.99	0.197
Male (%)	15/21 (71.4 %)	10/14 (71.4 %)	11/20 (55.0 %)	0.468
Duration (months)	12.21 ± 18.06	18.06 ± 30.99	30.20 ± 78.73	0.528
Follow-up time (months)	43.14 ± 23.90	53.43 ± 29.07	44.40 ± 24.01	0.467
Symptoms				
Dyspnea	15/21 (71.4 %)	13/14 (92.9 %)	19/20 (95 %)	0.067
Cough	14/21 (66.7 %)	10/14 (71.4 %)	16/20 (80 %)	0.627
Chest pain	2/21 (9.5 %)	1/14 (7.1 %)	1/20 (5 %)	0.856
Dry eyes or dry mouth	2/21 (9.5 %)	1/14 (7.1 %)	0	0.386
Fever	2/21 (9.5 %)	2/14 (14.3 %)	3/20 (15 %)	0.853
Arthralgia	6/21 (28.6 %)	3/14 (21.4 %)	1/20 (5 %)	0.138
Rash	5/21 (23.8 %)	1/14 (7.1 %)	0	0.044
Raynaud’s phenomenon	3/21 (14.3 %)	0	0	0.077
Weight loss	2/21 (9.5 %)	1/14 (7.1 %)	1/20 (5.0 %)	0.856
Signs				
Crackles	19/21 (90.5 %)	13/14 (92.9 %)	15/20 (75 %)	0.246
Clubbing	7/21 (33.3 %)	5/14 (35.7 %)	5/20 (25 %)	0.765

**Table 3:** Laboratory values of patients classified as CTD-NSIP, NSIP-Ab (+) and NSIP-Ab (−)

Laboratory tests	CTD-NSIP (*n* = 21)	Ab (+)-NSIP (*n* = 14)	Ab (−)-NSIP (*n* = 20)	*p* value
ESR, mm/h	16.33 ± 7.69	19.50 ± 12.70	27.45 ± 28.32	0.167
PaO_2_, mmHg	76.28 ± 14.25	80.67 ± 9.77	77.47 ± 13.52	0.614
PCO_2_, mmHg	40.20 ± 11.94	36.24 ± 5.57	36.74 ± 4.52	0.292
TLC, % predicted	77.17 ± 15.82	74.42 ± 10.37	77.01 ± 12.70	0.844
DLCO, % predicted	53.54 ± 14.06	56.62 ± 20.86	55.79 ± 8.60	0.893
Chest CT findings at biopsy				
Ground glass opacity	15/21 (71.4 %)	6/14 (42.9 %)	13/20 (65.0 %)	0.219
Patchy	16/21 (76.2 %)	10/14 (71.4 %)	14/20 (70.0 %)	0.899
Reticular opacity	12/21 (57.1 %)	11/14 (78.6 %)	12/20 (60.0 %)	0.397
Bronchiectasis	4/21 (19.0 %)	5/14 (35.7 %)	8/20 (40.0 %)	0.315
Pleural thickness	1/21 (4.8 %)	4/14 (28.6 %)	3/20 (15.0 %)	0.147

### Distribution of T lymphocyte subsets among different regions of lung tissue

**Fig. 2:**
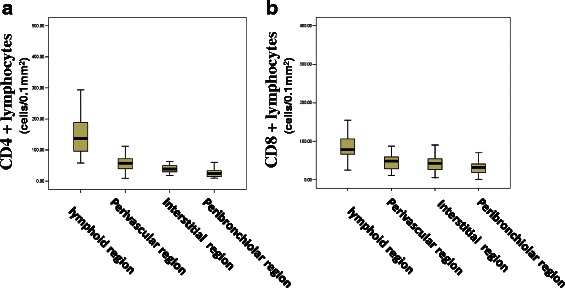
**a** Distribution of CD4+ T lymphocytes in different regions of lung tissue. The different in CD4+ cell counts between any two regions were statistically significant (*p* < 0.001). **b** Distribution of CD8 + T lymphocytes in different regions of lung tissue. CD8+ cell in follicles were much more than those in each of the other three regions (*p* < 0.001)

The distribution of CD4+ T lymphocytes is shown in Fig. 2A. The number of CD4+ T lymphocytes per 0.1 mm^2^ in the follicle, perivascular, interstitial, and peribronchial regions were 161.4 ± 89.0, 61.4 ± 30.6, 40.9 ± 12.0, and 25.9 ± 14.2, respectively. The numbers of CD4+ T cells decreased gradually in the order of the follicle, perivascular, interstitial, and peribronchial regions. The differences in cell counts between each of two different regions were statistically significant (*p* < 0.001).

The distribution of CD8+ T lymphocytes (Fig. 2B) was similar to that of CD4+ T lymphocytes. The numbers of CD8+ T lymphocytes per 0.1 mm^2^ in perivascular (47.6 ± 22.7), interstitial (45.7 ± 30.1), and peribronchial (32.5 ± 19.3) regions were not significantly different (*p* = 0.561). The number of lymphocytes in follicles (96.3 ± 58.4) were much greater than those in each of the other three regions (all *p* < 0.001).

### Distribution of CD4+ and CD8+ T lymphocytes among NSIP subtypes

We analyzed NSIP subtypes as follows: 22 cellular, 28 mixed, and five fibrotic. The distribution of CD4+ and CD8+ T lymphocytes in these subtypes is summarized in Table 4. Perivascular infiltration with CD4+ T lymphocytes was more prominent in patients with the cellular pattern compared with those with the mixed or fibrotic pattern. There was no significant difference in the number of infiltrating CD8+ T lymphocytes among the four anatomical regions.

**Table 4:** Distribution of T lymphocytes in patients with different histological patterns

Tissue	Cellular NSIP (*n* = 22)	Mixed NSIP (*n* = 28)	fibrotic NSIP (*n* = 5)	*p* value
CD4+ T lymphocytes (number/0.1 mm^2^)				
Follicle region	163.37 ± 89.58	159.69 ± 94.36	161.93 ± 68.14	0.990
Perivascular region	78.12 ± 34.79	49.74 ± 22.42	53.10 ± 18.17	0.003*
Interstitial region	40.85 ± 12.66	39.54 ± 11.52	38.48 ± 13.97	0.894
Peribronchial region	27.53 ± 15.60	25.70 ± 13.82	20.10 ± 8.84	0.575
CD8+ T lymphocytes (number/0.1 mm^2^)				
Follicle region	123.11 ± 77.81	72.62 ± 26.37	110.74 ± 36.94	0.006
Perivascular region	57.51 ± 23.43	38.91 ± 17.50	52.60 ± 29.89	0.011
Interstitial region	57.96 ± 39.81	36.26 ± 17.53	43.83 ± 18.69	0.037
Peribronchial region	39.11 ± 22.52	27.80 ± 16.27	29.40 ± 12.50	0.111

The numbers of CD4+ and CD8+ T lymphocytes cells in the CTD-NSIP (*n* = 21), NSIP-Ab (+) (*n* = 14), and NSIP-Ab (−) (*n* = 20) groups were not significantly different in each of the four anatomical regions (Table 5).

**Table 5:** Distribution of T lymphocytes in the CTD-NSIP, NSIP-Ab (+) and NSIP-Ab (−) groups

	CTD-NSIP group (*n* = 21)	NSIP-Ab (+) group (*n* = 14)	NSIP-Ab (−) group (*n* = 20)	*P* value
CD4+ T lymphocytes (number/0.1 mm^2^)				
Follicle region	181.5 ± 87.2	155.4 ± 110.7	144.4 ± 73.1	0.401
Perivascular region	70.2 ± 26.8	54.5 ± 25.4	51.2 ± 27.0	0.061
Interstitial region	42.9 ± 12.5	38.2 ± 10.9	38.15 ± 12.20	0.372
Peribronchial region	28.8 ± 13.4	25.6 ± 13.3	26.3 ± 16.1	0.989
CD8+ T lymphocytes (number/0.1 mm^2^)				
Follicle region	107.9 ± 72.2	102.4 ± 26.1	107.8 ± 50.9	0.139
Perivascular region	59.5 ± 23.7	46.6 ± 17.4	42.7 ± 19.8	0.115
Interstitial region	45.7 ± 23.9	33.3 ± 19.5	54.2 ± 39.0	0.139
Peribronchial region	36.0 ± 21.6	30.2 ± 15.7	30.4 ± 19.4	0.579

### T lymphocyte subsets in lung tissue and improved CT findings

**Fig. 3:**
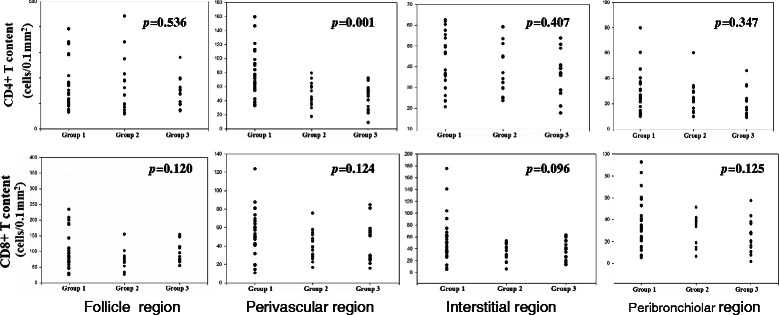
Correlation of T lymphocytes in different regions of tissue and prognosis of NSIP. Group 1: an improvement of ground-glass opacity and reticular opacity over 50 % on HRCT; Group 2: the lesions were still present but decreased between 30–50 % on HRCT; Group 3: the lesions were reduced less than 30 % on HRCT. There is statistical significance of CD4+ lymphocytes perivascular infiltrates among three groups (*p* = 0.001)

After undergoing therapy for 1 year, 25 patients (20 cellular and five mixed patterns) achieved a 50 % improvement in GGO and reticular opacity (CT-group 1). The lesions of 14 patients (all mixed pattern) decreased between 30 % and 50 % (CT-group 2). The lesions of 14 patients (nine mixed and five fibrotic patterns) were reduced by <30 % (CT-group 3). The number of CD4+ T lymphocytes in perivascular tissue in the CT-group 1 was significantly greater compared with those of other two CT-groups (76.5 ± 33.9 vs 49.5 ± 17.3, *p* = 0.009; 76.5 ± 33.9 vs 43.9 ± 18.4, *p* = 0.002) (Fig. 3), but not in the other anatomical regions. There was no correlation between the number of CD8+ T lymphocytes and the improvement HRCT findings.

### T lymphocyte subsets of lung tissue and pulmonary function tests

**Fig. 4:**
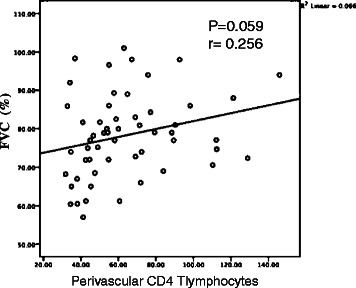
The relationship between perivascular CD4 infiltration and 12 months Follow-up FVC (*p* = 0.059, r = 0.256)

Cox multivariate regression analysis revealed that after 12 months of follow-up, FVC was an independent factor for survival (HR, 0.828; 95 % CI, 0.692–0.992; *p* = 0.040) (Additional file 1: Table S1). There was a marginal correlation of FVC with the number of perivascular CD4 cells (Fig. 4). Among 55 patients, 21 (11 cellular and 10 mixed patterns) achieved an improvement of FVC >10 % (FVC-group 1). No correlation between perivascular CD4 infiltration and DLCO at first visit (p=0.642, r=-0.064) (Additional file 2: Figure s2) and DLCO after 12 months follow up (p=0.134, r=-0.205) (Additional file 3: Figure S2).

**Fig. 5:**
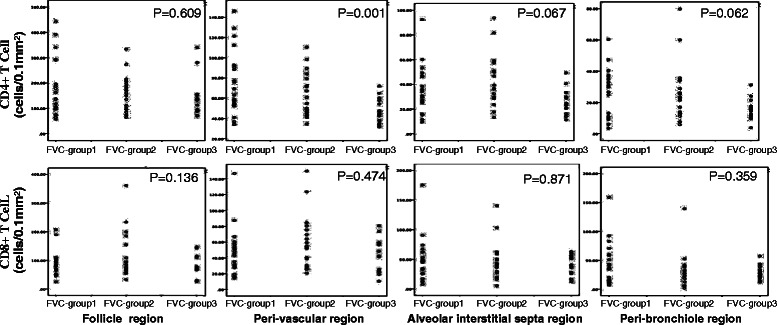
The relationship between CD4 T lymphocytes in perivascular regions of tissue and prognosis of FVC in NSIP patients. There are significant difference among three groups, *p* = 0.001.FVC-Group1 vs FVC-Group2 *p* = 0.056; FVC-Group1 vs FVC-Group3 *p* = 0.000; FVC-Group2 vs FVC-Group3 *p* = 0.054. FVC-group 1: an improvement of FVC over 10 % after 12 months treatment. FVC-group 2: FVC improved between 10 and −10 % after 12 months treatment. FVC-group 3: FVC worsen over −10 % after 12 months treatment

Nineteen patients (eight cellular, nine mixed, and two fibrotic patterns) achieved improved FVC between 10 % and −10 % (FVC-group 2). The FVC values of 15 patients (three cellular, nine mixed, and three fibrotic patterns) decreased greater than −10 % (FVC-group 3). The number of CD4+ T lymphocytes that infiltrated perivascular tissue in FVC-group 1 was significantly greater than those of the other two groups (77.9 ± 31.5 vs 63.0 ± 23.1, *p* = 0.056; 77.9 ± 31.5 vs 46.5 ± 12.5, *p* = 0.001) (Fig. 5).

The densities of CD4+ and CD8+ T lymphocytes T that infiltrated other anatomical compartments was not significantly associated with pulmonary function tests.

### The number of CD4+ T lymphocytes infiltrating perivascular tissue influences survival

Cox multivariate regression analysis considered age, sex, and numbers of CD4+ and CD8+ T lymphocytes infiltrating the lungs, and lung function tests (first visit and after 1 year of follow-up). The results revealed that perivascular infiltration of CD4+ T lymphocytes (HR, 0.939; 95 % CI, 0.883–0.999; *p* = 0.048) was an independent factor for survival (Additional file 1: Table S1).

**Fig. 6:**
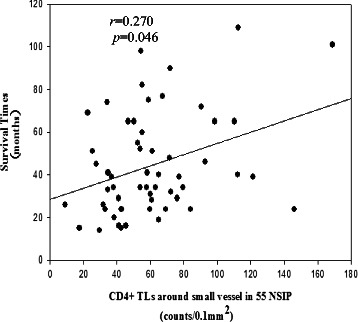
CD4+ T lymphocytes perivascular infiltration were closely related to survival time in NSIP patients. Correlation coefficients were calculated using the Spearman rank method

**Fig. 7:**
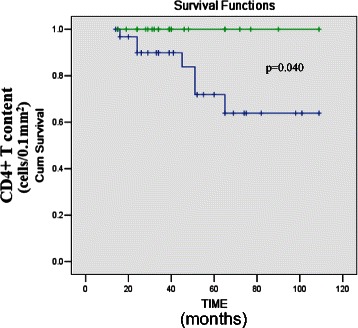
Kaplan-Meier survival curve for 55 NSIP patients grouped by the cell density of CD4 in small vessel region. Green line: ≥61 cells/0.1 mm2. Blue line: < 61 cells/0.1 mm2. There is a significant difference (log-rank test: 4.58, *p* = 0.04) between the groups

The densities CD4+ T lymphocytes infiltrating perivascular tissues of all patients were significantly associated with survival time (*r* = 0.270, *p* = 0.046) (Fig. 6) compared with those in other anatomical compartments. Further, patients with a much higher density of CD4+ T lymphocytes (≥61 cells/0.1 mm^2^) in the perivascular compartment survived longer (log-rank test, 4.58; *p* = 0.040) (Fig. 7). The cutoff value was determined using the ROC curve, and the AUC for patients’ survival was 0.79 (sensitivity 0.615, specificity 0.710).The densities of CD4+ and CD8+ T lymphocytes infiltrating other anatomical compartments were not significantly associated with survival time.

The most important finding of the present study was that the number of CD4+ T lymphocytes that infiltrated perivascular tissue correlated significantly with the prognosis of NSIP.

Immunohistochemical analyses revealed the T lymphocyte subsets infiltrated not only lymphoid follicles and interstitial regions, but perivascular and peribronchial regions as well, which is consistent with the findings of our previous study [7]. In this study, we did not detect a significant difference in T lymphocyte density among cellular, mixed, and fibrotic patterns among the same tissues analyzed here. The small number of patients with the fibrotic pattern might account for these findings, although we show here that there were more lymphocytes in patients with the cellular than with the fibrotic pattern. In contrast, in the present study, we found a correlation between the infiltration of perivascular tissue with CD4+ T cells with prognosis.

The predictors of prognosis of patients with ILD are the results of the pulmonary function test [18–20] and all-cause mortality [27–31]. In the present study, we combined them [29] to analyze perivascular CD4+ T lymphocyte infiltration and clinical prognosis. FVC consistently predicts the mortality of patients with IPF [17–20, 32] and is considered the best marker of chronic disease progression. FVC data now serve as the preferred primary endpoint in trials of therapies IPF [33].

In the present study, we show that FVC was an independent predictor of survival, which is consistent with the results of previous studies [32, 34–37]. When we classified patients according to the change of FVC after the 12-month follow-up, we found that higher numbers of perivascular CD4+ T lymphocytes correlated with improved FVC.

In the present study, all-cause mortality was 23.6 %. Cox multivariate regression analysis revealed that perivascular CD4+ T lymphocyte infiltration was an independent predictor of survival. Survival time correlated with the number of perivascular CD4+ T lymphocytes. Patients with higher numbers of perivascular T lymphocytes in the perivascular region have a better prognosis.

The accumulation of T lymphocytes in tissue is considered a cell-mediated immune reaction to bodily injury [38], particularly in patients with immune dysfunction [39]. The accumulation of perivascular CD4+ T lymphocytes occurs in patients with cutaneous lichen planus [40] and in the muscle tissue of patients with dermatomyositis [41]. Some studies found that T cells might be involved in the pathogenesis of interstitial lung disease. For example, the levels of CXCL9, CXCL10, and CXCL11 in bronchoalveolar lavage fluid indicate that lung fibroblasts induce a Th1-type immune response in patients with NSIP [42].

In an animal model of lung injury, perivascular CD4+ T lymphocytes accumulate in lung tissue [43, 44]. In patients with interstitial pneumonia caused by graft-versus-host disease, interstitial pneumonitis develops primarily from the dissemination of perivascular CD4+ T lymphocytes infiltrates [45]. Immunosuppressive agents such as cyclophosphamide suppress inflammation caused by perivascular T lymphocytes by reducing the T lymphocyte population and ameliorating T lymphocyte function [46]. These findings, taken together with those of the present study, suggest that perivascular infiltration of CD4+ T lymphocytes contributes to the pathogenesis of NSIP. If CD4+ T cells are involved in ILD, treating patients with anti-T cell-specific immunosuppressants such as FK506 and anti-TNF-α antibodies may be justified. For example, patients with ILD, particularly those with CTD-ILD, respond to these agents, [47, 48].

It is very unlikely that lung biopsies will be performed solely to count T-cells when pulmonary function tests and other less invasive measures are available. The implication of our findings related to therapy is that they provide an answer to the question of why anti-T cell treatment is beneficial for patients with ILD.

We recognize that our study is limited by its retrospective design, and B lymphocyte and other inflammatory cells were not investigated. However, to our knowledge, this is the first study to evaluate the relationship between perivascular infiltration of CD4+ T cells and the prognosis of patients with NSIP.

In conclusion, we show here that perivascular infiltration of CD4+ T cells correlated significantly with prognosis and survival. The underlying mechanisms require further study.

## Additional files

Additional file 1: **Table S1.** The prognostic factors study with multivariate analysis. (DOCX 406 kb)
Additional file 2: **Figure S1.** The relationship between perivascular CD4 infiltration and DLCO (*p* = 0.642, r = −0.064). (PPT 98 kb)
Additional file 3: **Figure S2.**The relationship between perivascular CD4 infiltration and 12 months follow-up DLCO (*p* = 0.134, r = −0.205). (PPT 43 kb)

## Abbreviations

CBC: Complete blood count
CTD: Connective tissue disease
DLCO: Diffusing capacity of the lung for carbon monoxide
ESR: Erythrocyte sedimentation rate
FVC: Forced vital capacity
HRCT: High-resolution computed tomography
ILD: Interstitial lung diseases
NSIP: Nonspecific interstitial pneumonia
TLC: Total lung capacity
