# Supplementary material for: Prognosis of nonspecific interstitial pneumonia correlates with perivascular CD4+ T lymphocyte infiltration of the lung
Source: BMC Pulm Med. 2015 Oct 24;15:127. doi: 10.1186/s12890-015-0122-z (PMC4619990; doi:10.1186/s12890-015-0122-z)
Supplement: Additional file 1: — Table S1. The prognostic factors study with multivariate analysis. (DOCX 406 kb) [file 12890_2015_122_MOESM1_ESM.docx]

Additional file 1: Table S1 The prognostic factors study with multivariate analysis

The prognostic factors were analyzed with multivariate analysis. The variables included year, sex, CD4 and CD8 cell infiltrating in the lung (small airway region, interstitial region, small vessel region and follicular region), lung function tests (including the first visit and after one year follow up). CD4 cells infiltrated in Peri-vascular region and FVC performed after 12 months are the independent factor for survival.

CD4AIR= CD4 cells infiltrated in Peri-bronchial region

CD4INT= CD4 cells infiltrated in Interstitial region

CD4VAS= CD4 cells infiltrated in Peri-vascular region

CD4 FOLLICU= CD4 cells infiltrated in lymphoid follicle region

CD8AIR= CD8 cells infiltrated in Peri-bronchial region

CD8INT= CD8 cells infiltrated in Interstitial region

CD8VAS= CD8 cells infiltrated in Peri-vascular region

CD8 FOLLICU= CD8 cells infiltrated in lymphoid follicle region

FEV1=performed before the surgical lung biopsy

FVC= performed after 12 months treatment

TLC= performed after 12 months treatment

DLCO= performed after 12 months treatment

FEV12= FEV1 performed after 12 months treatment

FVC2= FVC performed after 12 months treatment

TLC=TLC performed after 12 months treatment

DLCO=DLCO performed after 12 months treatment
