# Supplementary material for: Prognosis of nonspecific interstitial pneumonia correlates with perivascular CD4+ T lymphocyte infiltration of the lung
Source: BMC Pulm Med. 2015 Oct 24;15:127. doi: 10.1186/s12890-015-0122-z (PMC4619990; doi:10.1186/s12890-015-0122-z)
Supplement: Additional file 2: — Figure S1. The relationship between perivascular CD4 infiltration and DLCO (p = 0.642, r = −0.064). (PPT 98 kb) [file 12890_2015_122_MOESM2_ESM.ppt]

## Slide 1
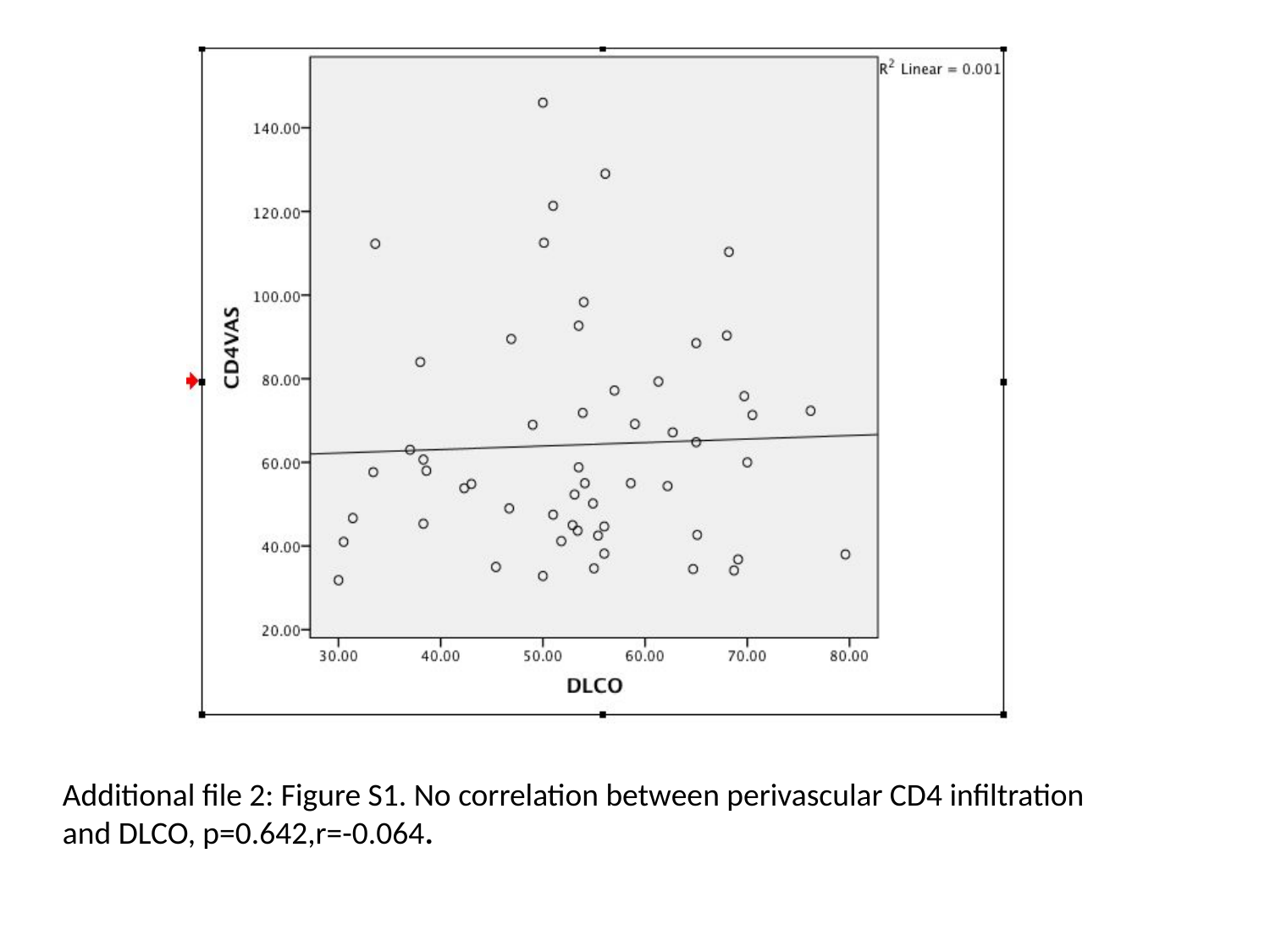

Additional file 2: Figure S1. No correlation between perivascular CD4 infiltration
and DLCO, p=0.642,r=-0.064.
